# Hypoxia-Inducible Factors: Mediators of Cancer Progression; Prognostic and Therapeutic Targets in Soft Tissue Sarcomas

**DOI:** 10.3390/cancers5020320

**Published:** 2013-04-02

**Authors:** Navid Sadri, Paul J. Zhang

**Affiliations:** Anatomic Pathology, Department of Pathology and Laboratory Medicine, Hospital of the University of Pennsylvania, 3400 Spruce Street, 6th Floor Founders Building, Philadelphia, PA 19104, USA; E-Mail: navid.sadri@uphs.upenn.edu

**Keywords:** soft tissue sarcoma, hypoxia, hypoxia-inducible factor, HIFs

## Abstract

Soft-tissue sarcomas remain aggressive tumors that result in death in greater than a third of patients due to either loco-regional recurrence or distant metastasis. Surgical resection remains the main choice of treatment for soft tissue sarcomas with pre- and/or post-operational radiation and neoadjuvant chemotherapy employed in more advanced stage disease. However, in recent decades, there has been little progress in the average five-year survival for the majority of patients with high-grade soft tissue sarcomas, highlighting the need for improved targeted therapeutic agents. Clinical and preclinical studies demonstrate that tumor hypoxia and up-regulation of hypoxia-inducible factors (HIFs) is associated with decreased survival, increased metastasis, and resistance to therapy in soft tissue sarcomas. HIF-mediated gene expression regulates many critical aspects of tumor biology, including cell survival, metabolic programming, angiogenesis, metastasis, and therapy resistance. In this review, we discuss HIFs and HIF-mediated genes as potential prognostic markers and therapeutic targets in sarcomas. Many pharmacological agents targeting hypoxia-related pathways are in development that may hold therapeutic potential for treating both primary and metastatic sarcomas that demonstrate increased HIF expression.

## 1. Introduction

Hypoxia is a common feature of many solid tumors owing to aberrant vascular function and rapid cell division. Neoplastic cells can survive under conditions of low oxygenation by activating adaptive responses to match oxygen supply with metabolic, bioenergetic, and redox demands [1]. Clinical and experimental studies have long demonstrated that tumor hypoxia is associated with increased malignancy, poor prognosis, and resistance to radiation and chemotherapy [2,3,4]. Patients with the most hypoxic soft tissue sarcomas have a worse disease-specific and overall survival [4] and increased likelihood of metastasis [5].

### 1.1. Hypoxia-Inducible Factors

The hypoxia-inducible factor (HIF) transcription factors mediate the primary transcriptional response to hypoxic stress in normal and neoplastic cells [6]. HIFs form heterodimeric complexes composed of an oxygen-liable α subunit and a stable β subunit. Together these subunits bind hypoxia response elements (HREs) on several hundred genes that facilitate the adaptation to hypoxia [6,7]. Mammals possess three isoforms of HIFα, of which HIF1α and HIF2α are the best characterized. HIF3α lacks the *C*-terminal transactivation domain and is believed to be a negative regulator of hypoxia-inducible gene expression, most likely by competing for binding with HIF1α and HIF2α in conditions where HIFβ is limited [8]. HIF1α is ubiquitously expressed, whereas HIF2α and HIF3α are selectively expressed in certain tissue types including vascular endothelium, liver parenchyma, and cells of myeloid lineage [2].

**Figure 1:**
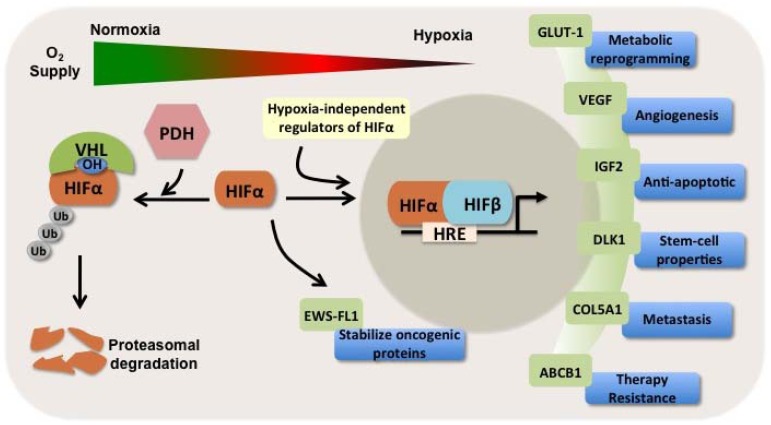
HIFs are involved in crucial aspects of tumor progression and metastasis in sarcomas. In the presence of oxygen, prolyl-hydroxylases (PDH) hydroxylate proline residues on HIFα subunit. Hydoxylated HIFα interacts with von Hippel-Lindau (VHL) protein, which is part of the E3 ubiquitin ligase complex that mediates the ubiquitination (Ub) of HIFα and targets it for proteasomal degradation. Under hypoxia, the α subunit is not hydroxylated. The stabilized α subunit moves into the nucleus, dimerizes with β subunit, and binds to hypoxia response elements (HRE) on target genes. HIFα is additionally up-regulated in a hypoxia-independent mechanism by several oncogenic pathways, including the PI3K-AKT-mTOR pathway. A representative HIF-regulated gene is shown to illustrate the importance of HIF target genes in many aspects of cancer biology in sarcomas. HIF-target genes are involved in: metabolic reprogramming, which include glucose transporter 1 (GLUT-1); induction of angiogenesis mediated by vascular endothelial growth factor (VEGF); promoting cell survival by encoding insulin-like growth factor 2 (IGF2); enhance stem-cell self-renewal ability through expression of delta-like 1 (DLK-1); promote metastasis through regulation of extracellular matrix genes, such as collagen, type V, α1 (COL5A1); and chemotherapy resistance through expression of ATP-binding cassette transporter B1 (ABCB1) that efflux chemotherapy drugs from cancer cells. In addition, HIFs indirectly regulate gene and protein expression by transactivation of chromatin-modifying genes and microRNAs. As an example, HIF1α up-regulates EWS-FL1 onco-protein expression and modulates its transcriptional signature towards metastasis-related genes.

HIF activity is controlled primarily through the stabilization of HIF1α and HIF2α protein subunits, which increases as cells become more hypoxic. HIFα subunits are modified by hydroxylation of two proline residues by HIF-specific prolyl-hydroxylases (PDHs) in the presence of oxygen, which leads to normoxic proteasomal degradation that is in part mediated by the von Hippel-Lindau (VHL) tumor suppressor protein [6] (Figure 1). Furthermore, under normoxic conditions, HIF1α is hydroxylated at residue Asn803 by factor inhibiting HIF1 (FIH), which disrupts a critical interaction between HIFα and coactivator p300, blocking HIF1-dependent transcriptional activition [1]. In the context of increased oncogenic signaling, HIF1α expression is also increased in cancer cells by hypoxia-independent mechanisms that include increased transcription and/or translation of HIF1α mRNA, as well as, increased protein stability [1]. Activated phosphoinositide-3 kinase (PI3K)-AKT-mammalian target of rapamycin (mTOR) signaling, a common feature of many cancer cells, increases the rate of HIF mRNA translation, leading to increased HIF expression and activity [9].

### 1.2. HIFs and Soft Tissue Sarcoma

Each year, soft tissue sarcomas arise in over 12,000 persons in the United States, and 35% of patients die of either loco-regional recurrence or distant metastasis [10]. Like other solid tumors, as sarcomas outgrow their blood supply, hypoxia stabilizes HIF1α and HIF2α, which bind to HIF-β (ARNT), and drive the transcription of over 150 genes crucial in many aspects of cancer biology including angiogenesis, epithelial-mesenchymal transition, stem-cell maintenance, invasion, metastasis, and resistance to radiation therapy and chemotherapy [11] (Figure 1). Based on genome-wide chromatin immunoprecipitation studies the estimated number of direct HIF target genes is greater than 800 genes [8]. HIFs also indirectly regulate gene expression by transactivation of chromatin-modifying genes and microRNAs [7,12,13]. Hierarchical clustering analysis using expression data from >100 hypoxia-related genes on oligonucleotide microarrays of sarcomas and normal tissues identified distinctly different patterns of expression; numerous hypoxia-related genes were significantly up-regulated in sarcomas including HIF1α [14].

The importance of hypoxia in many human cancers has prompted intensive research into understanding the mechanisms by which hypoxic tumor cells alter their transcriptional profiles to modulate critical pathways important in cancer biology [1,6]. There is also a growing body of preclinical evidence supporting the importance of the HIF pathway in sarcoma progression, metastasis, and therapy resistance. Only recently has the use of HIFs and HIF-targets as prognostic markers and potential therapeutic targets in soft tissue sarcomas been explored. 

### 1.3. HIFs as Independent Prognosticators in Soft Tissue and Bone Sarcomas

There is compelling evidence that activation of the HIF pathway promotes oncogenesis and cancer progression, including clinical data showing an association between increased HIF expression and decreased patient survival in many human cancers, including sarcomas [15,16,17,18,19,20,21]. Several reports have shown that hypoxia and elevated HIFs expression are correlated with a significantly shorter overall survival and metastasis free survival in soft tissue sarcomas, and in certain studies irrespective of histological diagnosis [4,22,23,24,25]. As an example, increased expression of HIF1α and HIF2α in chondrosarcomas is associated with worse patient survival [18,19]. HIF1α and HIF2α up-regulation play a prominent role in evasion of apoptosis and tumor progression associated with high Bcl-xL and low Beclin 1 expression in chondrosarcomas [18,19]. Whereas the trend from these studies is that HIF1α and HIF2α promote cancer progression, the association is not absolute. In one report, high expression of HIF1α mRNA in certain sarcomas was correlated with a significantly more favorable prognosis [26]. This may suggest differences in transcriptional and post-transcriptional mechanisms of HIF mRNA and/or may highlight complex and even opposing function by different isoforms of HIF during different stages of tumor development.

## 2. HIFs: Mediators of Sarcoma Progression, Metastasis, and Therapy Resistance

### 2.1. HIFs as Regulators of Sarcomagenesis

Early data showing that HIF1α expression was correlated with aberrant p53 accumulation and cell proliferation in various solid tumors and their metastasis indicated the important role that HIFs may play in human cancer progression [27]. Although the pathogenesis of sarcoma subtypes varies greatly, HIF-regulated genes have been shown to be important in the pathobiology of various sarcoma subtypes.

#### 2.1.1. Rhabdomyosarocoma

Several lines of evidence suggest a role HIF1α in rhabdomyosarcoma tumorigenesis. Under hypoxia, glucose transporter 1 (GLUT-1) is induced in a HIF1α-dependent manner increasing glucose uptake and playing an important role in conferring apoptosis resistance [28]. Hypoxia also enhances the stem-like population of cells within rhabdomyosarcoma cell lines [29]. More recently, pharmacological targeting of the HIF1α signaling pathway has been shown to inhibit rhabdomyosarcoma growth in xenograft models [30]. 

#### 2.1.2. Kaposi’s Sarcoma

As the master regulator of the hypoxic vascular response, it should not be surprising that HIFs plays a central role in Kaposi’s sarcomagenesis (KS) [31]. HIF1α drives transcriptional activation of hundreds of genes involved in vascular reactivity, angiogenesis, arteriogenesis, and the recruitment of endothelial precursor cells, all key steps toward the development of KS [31,32]. The latency-associated nuclear antigen (LANA) encoded by Kaposi’s sarcoma-associated herpesvirus (KSHV) is critical for nuclear accumulation of HIF1α in normoxia as it targets the HIF1α suppressors von Hippel-Lindau protein and p53 for degradation [33,34]. KSHV G protein-coupled receptor (vGPCR) upregulates vascular endothelial growth factor (VEGF) in endothelial cells through an mTOR-dependent increase in HIF1α and HIF2α protein levels [32]. Pharmacologic inhibition of HIFs blocked VEGF secretion and lead to regression of tumor allografts in this model [32].

#### 2.1.3. Gastroinstestinal Tumor

In a subset of “wild-type” gastrointestinal tumors (GISTs) with succinate dehydrogenase loss-of-function, it is postulated that elevation of succinate levels negatively regulates prolyl hydroxylase leading to increased HIF1α levels [35]. In line with this model, wild-type GISTs show increased expression of VEGF and insulin-like growth factor 2 (IGF2), HIF1α transcriptional targets, as compared to KIT-mutated GISTs [36]. IGF2 may activate IGFR in an autocrine manner resulting in increased signaling through the PI3K-AKT pathway [35]. 

#### 2.1.4. Liposarcoma

Several lines of evidence suggest HIF1α involvement in the progression of liposarcoma to a dedifferentiated state. In synchronous liposarcoma lesions that contain both well-differentiated component (adipocyte-like differentiation) and dedifferentiated component (lacking adipocyte differentiation and frequently showing other mesenchchymal differentiation), HIF1α is primarily detected in the dedifferentiated component [24]. Hypoxia has been shown to inhibit adipocyte differentiation through the repressive activity of HIF1α-induced differentiated embryo-chondrocyte expressed gene 1 (DEC1) on *PPARγ2* expression [37]. Furthermore, hypoxia via an HIF-dependent mechanism promotes the maintained expression of delta-like 1 (DLK1), a key stem cell gene that negatively regulates adipogenic differentiation and may facilitate the maintenance and/or selection of cancer cells with stem cell properties [38,39]. 

#### 2.1.5. Ewing’s Sarcoma

In Ewing’s sarcoma cells, EWS-FLI1 protein expression is upregulated by hypoxia in a HIF1α-dependent manner [40]. Furthermore, hypoxia modulates the EWS-FLI1 transcriptional signature towards the expression of metastasis-related genes and leads to invasiveness and soft agar colony formation *in vitro* [40]. Although HIF1α and HIF2α were previously suspected to have overlapping functions, more recent data suggest isoform-specific transcriptional responses. Experiments in Ewing’s sarcoma and osteosarcoma cell lines highlight isoform-specific HIF transcriptional response to hypoxia and hypoglycemia [41]. Downstream transcription of transcripts containing the VEGF and GLUT-1 hypoxia-response element (HRE) was HIF1α-dependent in Ewing’s sarcoma, but regulated by both isoforms in osteosarcoma [41]. The specific mechanism(s) whereby HIF promotes Ewing’s sarcoma and osteosarcoma progression *in vivo* remains to be identified. Furthermore, future studies need to better define isoform-specific transcriptional responses and function in an oncogenic context.

In certain contexts, hypoxia alone may not be enough to activate the HIF system. Despite the presence of extreme hypoxia, HIF1α is not up-regulated in benign uterine leiomyomas, however in their malignant counterparts, leiomyosarcomas, show a strong induction of the HIF system [42]. The authors suggest the strong activation of the HIF system observed in solid malignant tumors may be mechanistically linked to their transformed phenotype, rather than being a physiological reaction activated in a pathological context [42].

### 2.2. HIFs as Regulators of Metastasis

It has been shown that tumor oxygenation predicts the likelihood of distant metastases in human sarcomas [5,43,44]. Gene expression data from human tumors and work with experimental mouse models highlight the importance of HIF pathway activation in sarcoma metastasis. In a genetically engineered, temporally and spatially restricted, mouse model of pleomorhpic undifferentiated sarcomas, the HIF-target FOXM1 is highly associated with lung metastasis [44]. Gene expression microarray analysis in a group of 177 sarcomas revealed a prognostic profile of hypoxia-related genes predictive of metastatic potential in high grade, pleomorphic, genetically-complex sarcomas [43]. A separate gene expression microarray analysis suggested the existence of at least two subsets of high-grade pleomorphic STS with distinct clinical behavior, with tumors with increased metastatic propensity showing increased expression of HIF-dependent extracellular matrix genes, including COL5A1, COL1A2, and PLOD2 [45]. Independent of currently used prognosticators, these results support that hypoxia-related gene expression signature provide diagnostic utility in improved selection of high-risk STS patients.

Studies from other cancer types suggest that metastasis is achieved through a stepwise selection process driven by hypoxia [46]. HIF1α-dependent up-regulation of cathepsin D, urokinase-type plasminogen-activator receptor, and matrix metalloproteinase-2 enable cellular invasion through the basement membrane and the underlying stroma [47]. Studies in breast and head and neck cancers have shown that hypoxia-induced lysyl oxidase (LOX) is essential for tumor metastasis as LOX covalently modifies collagens to increase focal adhesion kinase activity, cell migration, and metastasis [48]. Hypoxia-induced VEGF promotes intravasation and extravasation by helping to increase microvascular permeability and interstitial fluid pressure [46]. ANGPTL4, a key molecule for extravasation to the lung, is up-regulated by HIF1α [49]. Hypoxia may increase metastatic homing by inducing chemokine receptor CXCR4, which plays a key role in metastatic homing of tumor cells to organs expressing high level of its ligand, SDF1 [50]. LOX also acts as a critical mobilizing factor, which recruits CD11b+ myeloid cells to form the niche to facilitate the colonization of metastatic tumor cells [51]. Through regulation of these critical molecular targets, HIFs promote various steps of the metastatic cascade and provide an adaptive advantage to select tumor cell populations to survive and escape the unfavorable microenvironment of the primary tumor [46]. It is currently unclear if sarcoma cancer cells use similar molecular mechanisms or employ other HIF-dependent pathways to achieve metastatic potential.

Hypoxia may also influence organ-specific metastasis. In breast cancer, hypoxia enhances the expression of a large percentage of genes involved in lung metastasis, while it activates a more limited number in bone metastasis [27]. The role of HIFs in organ-specific metastasis needs to be better studied in sarcomas, especially with respect to lung metastasis that constitute the majority of metastatic diseases. Future studies need to address if HIF-targets that promote survival, metabolic reprogramming, invasion and angiogenesis in the primary tumor function similarly in secondary sites. 

### 2.3. HIFs as Regulators of Therapy Resistance

It is well established that hypoxic cells within a tumor limit the effectiveness of radiotherapy, with the requirement of free oxygen to covert free radicals initiated by ionizing radiation to form DNA strand breaks [52]. HIFs also contribute to radiation resistance. Inhibition of HIF-2α leads to tumor cell death and enhances the response to radiation treatment [53]. HIF1α also promotes tumor radioresistance through stimulation of endothelial cell survival [54]. In a phase II clinical trial, neoadjuvant bevacizumab, an anti-VEGF therapy, can significantly augment the therapeutic efficacy of radiation therapy against soft tissue sarcomas and may reduce the incidence of local recurrence [55].

A contribution of HIF1α to chemoresistance of neoplastic cells has been observed in a wide spectrum of solid tumors, including fibrosarcoma [56,57,58]. Clinically, HIF1α has been shown to be an independent factor for resistance to chemotherapy in several solid tumor types [59,60,61]. The mechanisms underpinning HIF1α-mediated chemoresistance need to be better studied in the heterogenous group of sarcomas that exhibit resistance to therapy. Furthermore, HIF1 activity may contribute to resistance to more targeted therapies, such as imatinib, through metabolic reprogramming [62].

## 3. Agents Targeting HIFs

Several approaches targeting hypoxia in sarcoma cells have been explored, including the use of bioreductive prodrugs that are converted to cytotoxins under hypoxic conditions, developing inhibitors of HIF1 expression and activity, targeting oncogenic pathways (mTOR) regulating HIF1 expression, and targeting specific HIF downstream pathways [63]. Most of these agents have previously been reviewed elsewhere [11,63,64,65], and will not be extensively covered here.

Bioactive prodrugs, agents that only become activated in oxygen-poor conditions, are designed to exploit the hypoxic microenvironment in many sarcomas. TH-302, a nitroimidazole prodrug of the DNA alkylating agent, bromoisophosphoramide mustard (Br-IPM), is reduced in hypoxic conditions leading to release of the Br-IPM and DNA cross-linking [66]. TH-302 has minimal cytotoxic activity under normoxic conditions, minimizing systemic toxicity seen with ifosfamide, a similar systemic cross-linking agent. The standard single-agent chemotherapy for metastatic or locally advanced unresectable disease is doxorubicin, with an expected response rate of 12–23% and median progression-free survival of 4–6 months [66]. Updated results of phase 2 clinical trial involving 91 metastatic or locally advanced unresectable STS reported at 2012 Connective Tissue Oncology Society meeting in Prague showed that TH-302 boosted the response rate to 36% with a median progression-free survival of 6.7 months [66,67]. In March 2012, TH-302 was granted orphan drug status in the United States and Europe to treat soft-tissue sarcoma [68]. Given the marked heterogeneity in tumor hypoxia, there is a great need for the development of diagnostics to better predict patient stratification and sensitivity as new drugs are discovered [63].

Agents targeting HIFs are in various stages of clinical development [69]. Currently, there is a growing number of chemical compounds that have been shown to block xenograft growth and inhibit HIF activity through a variety of molecular mechanisms such as decreased mRNA expression, protein synthesis and stabilization, subunit dimerization, DNA binding, and transcriptional activity of HIFs [11]. Antisense oligonucleotide (EZN2968) [70], and the aryl hydrocarbon receptor ligand aminoflavone have been shown to reduce HIF1α mRNA levels [71]. Several agents reduce HIF1α mRNA translation, including topoisomerase I inhibitors, such as EZN2208 [72], and mTOR inhibitors, temsirolimus and ridaforolimus [30,73]. HIF1α protein stability can be targeted by 17DMAG, an inhibitor of the chaperone heat sock protein 90 (Hsp90) [74]. Although most published studies correspond to the regulation of HIF1α, many of these agents might also affect HIF2α expression and activity [64]. 

Other HIF inhibitors have been discovered through phenotypic screens. A screen of drugs in clinical trials and/or use revealed that digoxin and other cardiac glycosides inhibit HIF1α protein synthesis and expression of HIF1α target genes in cancer cells [65]. In tumor xenografts, administration of digoxin increased latency and decreased growth, whereas treatment of established tumors resulted in growth arrest within one week [65]. Another screen using a cell-based reporter gene assay identified the anthracycline chemotherapeutic agents doxorubicin and daunorubicin as potent inhibitors of HIF1α-mediated gene transcription by blocking HIF binding to DNA [75].

Given the prominent role of mTOR in control of HIF expression, several mTOR inhibitors are under clinical investigation for patients with STS [68]. A large prospective study of ridaforolimus, an mTOR inhibitor, in 212 patients with metastatic or unresectable sarcomas showed a clinical benefit rate of 29% [73], which is greater than the expected rate with standard doxorubicin therapy [66]. Patients in the leiomyosarcoma and liposarcoma cohorts had slightly higher clinical benefit rates than those patients in the “other” cohort, highlighting the need for better biomarkers to identify patients who may respond [73]. A randomized, placebo-controlled Phase III study of ridaforolimus as maintenance therapy in 711 patients with advanced bone and soft tissue sarcomas who had at least stable disease following prior chemotherapy, showed an improvement in progression free survival as compared to the placebo group [73,76,77].

In addition, combining several HIF inhibitors may allow lower doses of individual agents, thereby reducing the likelihood of off-target effects, while still allowing for effective inhibition of the HIF pathway by multiple mechanisms [78]. In addition, combination treatment with HIF inhibitors may improve the efficacy of anti-VEGF therapy, by blocking compensatory pathways exploited by cancer cells to overcome environmental stresses [78]. To expand the use of agents targeting VEGF and HIFs in sarcoma patients, it is essential to understand better the complex role of HIFs in controlling sarcoma progression and metastasis and the biological effects of VEGF and HIF inhibition on chemo- and radiosensitivity in these tumors.

Currently, there is insufficient data to identify specific STS subtypes or specific class of tumors within a subtype that are more likely to benefit from hypoxia-driven therapies. Although there are no validated predictive biomarkers for the response to hypoxia-target therapy, future research should lead to selection of patients more likely to respond [66]. Furthermore, molecular imaging techniques, such as positron emission tomography imaging with fluorinated nitroimidazoles, are being developed to better predict the response to hypoxia-target therapies [79].

## 4. Conclusions

Mounting evidence suggests that hypoxia-induced factors are important regulators in tumor progression, metastasis, and therapy resistance in soft tissue sarcomas. HIFs and HIF-mediated genes can be used as important prognosticators to stratify patients with sarcomas into groups with distinct clinical behavior improving selection of high-risk cancers for management. For their critical role in regulating sarcoma progression, HIFs and HIF-mediated genes may provide a brand new frontier to fight sarcoma. Better understanding of HIFs in regulating key oncogenic pathways will expand the use of agents targeting hypoxia-related genes in sarcoma patients. Pharmacological agents are being developed to target hypoxia-related pathways in human cancers, and this growing library of agents may hold therapeutic implications for treating both the primary sarcoma and metastatic disease. Future studies utilizing more accurate tumor models should tease out the effects of targeting the HIF pathway because different isoforms of HIF are likely to have complex and even opposing functions during different stages of tumor development. In addition, better molecular and imaging markers are needed to improve patient selection and treatment-response surveillance to parallel the use of HIF inhibitors.
